# Comparison of Complications after Coronavirus Disease and Seasonal Influenza, South Korea

**DOI:** 10.3201/eid2802.211848

**Published:** 2022-02

**Authors:** Hyejin Lee, Ho Kyung Sung, Dokyoung Lee, Yeonmi Choi, Ji Yoon Lee, Jin Yong Lee, Myoung-don Oh

**Affiliations:** Seoul National University Bundang Hospital, Seongnam-si, South Korea (H. Lee); National Medical Center, Seoul, South Korea (H.K. Sung, M. Oh);; Health Insurance Review & Assessment Service Research Institute, Wonju-si, South Korea (D. Lee, Y. Choi, J.Y. Lee, J.Y. Lee);; Seoul National University Hospital, Seoul (J.Y. Lee); Seoul National University College of Medicine, Seoul (J.Y. Lee, M. Oh)

**Keywords:** COVID-19, SARS-CoV-2, severe acute respiratory syndrome coronavirus 2, influenza, viruses, respiratory infections, zoonoses, complications, long COVID, coronavirus disease, *Suggested citation for this article*: Lee H, Sung HK, Lee D, Choi Y, Lee JY, Lee JY, et al. Comparison of complications after coronavirus disease and seasonal influenza, South Korea. Emerg Infect Dis. 2022 Feb [*date cited*]. https://doi.org/10.3201/eid2802.211848

## Abstract

We conducted a retrospective cohort study using claims data to determine the number and types of complications from coronavirus disease (COVID-19) that patients experience and which patients are more vulnerable to those complications compared with complications in patients with influenza. Among the cohort, 19.6% of COVID-19 patients and 28.5% of influenza patients had >1 new complication. In most complications, COVID-19 patients had lower or similar relative risk compared with influenza patients; exceptions were hair loss, heart failure, mood disorder, and dementia. Young to middle-aged adult COVID-19 patients and patients in COVID-19 hotspots had a higher risk for complications. Overall, COVID-19 patients had fewer complications than influenza patients, but caution is necessary in high-risk groups. If the fatality rate for COVID-19 is reduced through vaccination, management strategies for this disease could be adapted, similar to those for influenza management, such as easing restrictions on economic activity or requirements for close-contact isolation.

The symptoms, incidence, and risk for complications of coronavirus disease (COVID-19) remain controversial. Several types of long COVID, meaning prolonged symptoms or long-term complications of COVID-19, have been reported (*1*). In addition to fibrosis and decreased pulmonary function from inflammation of the lungs (*2*), other long-term complications have been reported as well (*3*–*7*). In one study, >1 symptoms remained in 87.4% of COVID-19 patients at a mean duration of 2 months after infection; fatigue and dyspnea were most common (*3*). Neurologic complications, such as loss of smell or taste, have also been reported (*4*,*5*). Moreover, unlike in severe acute respiratory syndrome (SARS) and Middle East respiratory syndrome (MERS), cerebral vascular disease has been reported to be more common in COVID-19. Ischemic stroke, hemorrhage, and cerebral venous sinus thrombosis have been reported in 2%–6% of hospitalized patients with COVID-19 (*6*), and it has been estimated that cognitive impairment and dementia associated with cerebral vascular disease will increase (*7*).

The National Institute for Health and Care Excellence in the United Kingdom recently defined long COVID as signs and symptoms that develop during or after an infection consistent with COVID-19 that continue for >4 weeks and cannot be explained by an alternative diagnosis (*8*). Long COVID is classified according to duration of signs and symptoms: those lasting 4–12 weeks are classified as ongoing symptomatic COVID-19, and those lasting >12 weeks are defined as post–COVID-19 syndrome.

Many patients report long COVID, and the need for management is high; however, definitive identification of symptoms, characteristics of vulnerable patients, and risk for COVID complications remain elusive. One study compared the illness and death rates of hospitalized patients for COVID-19 and influenza but did not include nonhospitalized COVID-19 or influenza patients with mild illness (*9*). In this study, we aimed to investigate the number and type of COVID-19 complications that occur and which COVID-19 patients are more vulnerable to complications compared with influenza patients. The Institutional Review Board (IRB) of the Health Insurance Review and Assessment Service approved this study (IRB no. 2021087-001).

## Methods

### Study Design, Setting, and Population

We conducted a retrospective cohort study using claims data provided by the Health Insurance Review and Assessment Service (HIRA). South Korea has adopted mandatory universal health coverage; therefore, 97% of South Korea residents are National Health Insurance Service (NHIS) beneficiaries and pay NHIS premiums according to their income levels or property values. The remaining 3% are medical-aid (MA) recipients who are unable to pay premiums; their medical costs are covered by the government through taxes (*10*). Therefore, medical use, diagnosis, and treatment history of COVID-19 and influenza patients can all be identified from claim data. The diagnosis code was based on the Korean Standard Classification of Diseases and Causes of Death, 7th edition (KCD-7), which is a modification of the International Classification of Diseases, 10th Revision.

We defined COVID-19 patients as those who received diagnosis of and were treated for COVID-19 (KCD-7 code U07.1); we enrolled a total of 21,615 patients during January 1–September 30, 2020. We defined influenza patients as those who were prescribed the antiviral drugs oseltamivir, zanamivir, or amantadine for an influenza-like illness (*11*) or who had influenza diagnosed (KCD-7 code J09-J11) as determined by a doctor; we enrolled 2,380,696 patients during July 1, 2017–June 30, 2018. We defined complications for COVID-19 and influenza patients as signs or symptoms in patients who had not received a diagnosis of those specific conditions in the last 3 years but who received new diagnoses of complications during the follow-up period after COVID-19 or influenza diagnosis.

**Figure 1:**
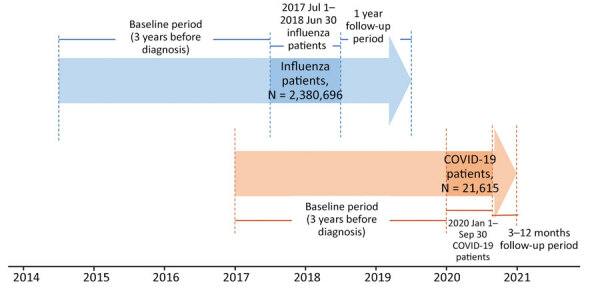
Study design for comparison of complications in coronavirus disease and influenza patients, South Korea. COVID-19, coronavirus disease.

We followed influenza patients for up to 1 year after diagnosis (follow-up duration 365 days) and COVID-19 patients from the date of COVID-19 diagnosis to December 31, 2020 (median follow-up duration 209 days; interquartile range 127–297 days) (Figure 1).

### Data Collection

We extracted patient sex, age, insurance type, region, Charlson Comorbidity Index (CCI), severity and complications from the claims data. We defined age groups as 0–19 years, 20–44 years, 45–64 years, 65–74 years, and >75 years of age. We categorized insurance as NHIS beneficiaries and MA recipients. We classified regions into the Seoul metropolitan area, Daegu and Gyeongsangbuk Province, and other areas, in accordance with the COVID-19 epidemic areas in South Korea. We calculated the CCI, an indicator of underlying conditions, using claims data from 1 year before the diagnosis of COVID-19 or influenza. 

We classified severity as ambulatory, hospitalized mild disease, and hospitalized severe disease using the COVID-19 severity scale from the World Health Organization (*12*). The scale indicates the clinical severity of the disease and can be used during any infectious disease epidemic; therefore, influenza patients were classified on the same scale (*13*). The complications we used were gastrointestinal disease, musculoskeletal disorder, periodontal disease, dermatitis, hair loss, asthma, chronic obstruction pulmonary disease, pneumonia, cardiovascular disease, heart failure, cerebrovascular disease, autoimmune disease, mood disorder, and dementia. Complications were confirmed with the KCD-7 code (Appendix Table 1).

**Table 1:** Baseline characteristics of participants in study comparing complications of COVID-19 and influenza, South Korea*

Characteristic	General population†	COVID-19	Influenza	p value‡
Total no. participants	52,870,968	21,615	2,380,696	
Sex				0.14
M	26,455,169 (50.0)	9,700 (44.9)	1,080,288 (45.4)	
F	26,415,799 (50.0)	11,915 (55.1)	1,300,408 (54.6)	
Age, y				<0.001
0–19	8,822,808 (16.7)	1,467 (6.8)	1,180,279 (49.6)	
20–44	18,223,124 (34.5)	7,665 (35.5)	531,254 (22.3)	
45–64	17,344,828 (32.8)	7,841 (36.3)	462,183 (19.4)	
65–74	4,907,575 (9.3)	2,755 (12.8)	11,5673 (4.9)	
≥75	3,572,633 (6.8)	1,887 (8.7)	9,1307 (3.8)	
Type of insurance				<0.001
Health insurance	51,344,938 (97.1)	20,218 (93.5)	2,327,645 (97.8)	
Medical aid	1,526,030 (2.9)	1,397 (6.5)	53,051 (2.2)	
Region				<0.001
Seoul metropolitan area	26,724,640 (50.5)	12,386 (57.3)	1,070,695 (45.0)	
Daegu and Gyeongsangbuk province	5,111,575 (9.7)	4,963 (23.0)	256,833 (10.8)	
Other areas	21,034,753 (39.8)	4,266 (19.7)	1,053,168 (44.2)	
Charlson Comorbidity Index score				<0.001
0	NA	13,467 (62.3)	1,533,247 (64.4)	
1–2	NA	6,484 (30.0)	755,602 (31.7)	
>3	NA	1,664 (7.7)	91,847 (3.9)	
Severity	NA			<0.001
Ambulatory state	NA	5,224 (24.2)	2,151,801 (90.4)	
Hospitalized mild disease	NA	15,447 (71.5)	222,572 (9.4)	
Hospitalized severe disease	NA	944 (4.4)	6,323 (0.3)	

*Values are no. (%) patients except as indicated. COVID-19, coronavirus disease; NA, not applicable. †Data source: 2020 National health insurance statistics. ‡χ^2^ tests were performed to assess the difference in proportion between COVID-19 and influenza patients.

### Statistical Analysis

We analyzed characteristics of the general population, COVID-19 patients, and influenza patients by frequency and percentage. We determined homogeneity by χ^2^ test. We determined the number of patients with complications by χ^2^ test and calculated the age-standardized incidence rate as the number of patients with complications divided by the sum of the total person-months by age, standardized by the population in 2020. The censoring time of the observation period was at the time when complications occurred or at the time of the last observation (1 year after influenza diagnosis; December 31, 2020, for COVID-19). The incidence of COVID-19 complications compared with influenza complications is a rate ratio. We performed multivariable logistic regression analysis to identify factors related to COVID-19 complication occurrence. We defined significance level as a 2-tailed p-value <0.05. We used SAS Enterprise guide version 7.15 (SAS Institute Inc., https://www.sas.com) for all analyses.

## Results

We reviewed a total of 21,615 cases of COVID-19 from January 1–September 30, 2020, and 2,380,696 cases of influenza from July 1, 2017–June 30, 2018. We report results by study characteristic: patient sex, age, insurance type, region, CCI, severity, and complications.

The sex ratio (M:F) was similar to that of the general population in both the COVID-19 and influenza patient groups. In the COVID-19 patient group, the number of patients 0–19 years of age was 1,467 (6.8%), whereas that age group was the largest of the influenza patient groups at 1,180,279 (49.6%). A total of 1,397 (6.5%) of MA recipients were COVID-19 patients, which was higher than for the general population (1,526,030, 2.9%) and for influenza patients (53,051, 2.2%) (p<0.001). A total of 4,963 (23.0%) COVID-19 patients lived in the provinces of Daegu and Gyeongsangbuk, hotspots of the pandemic; there was no regional influenza epidemic of influenza. A CCI score >3 was more common in COVID-19 patients (7.7%, 1,664) than in influenza patients (3.9%, 91,847). More COVID-19 patients than influenza patients were hospitalized with mild cases (71.5% vs. 9.4%) and severe cases (4.4% vs. 0.3%) (Table 1).

Among all COVID-19 patients, 4,139 (19.1%) had >1 complications after their diagnosis, and 678,845 (28.5%) of influenza patients had >1 complications after their diagnosis; thus, significantly more complications occurred in influenza patients (p<0.001). Skin disease (5.4% vs. 11.3%; p<0.001), asthma (0.5% vs. 3.1%; p<0.001) and pneumonia (2.1% vs. 4.4%; p<0.0001) were more prevalent in influenza patients than in COVID-19 patients. When the age-standardized incidence rate was calculated after standardization to the population in 2020, most of the COVID-19 patient group had a lower or similar relative risk (RR) of complications than the influenza group. However, COVID-19 patients had higher RRs for hair loss (RR 1.52, 95% CI 1.18–1.97), heart failure (RR 1.88, 95% CI 1.42–2.50), mood disorder (RR 1.73, 95% CI 1.56–1.93), and dementia (RR 1.96, 95% CI 1.52–2.55)(Table 2).

**Table 2:** Frequency and incidence rate of complications among COVID-19 and influenza patients, South Korea*

Complications	Frequency†				Incidence‡		Rate ratio (95% CI)§
	COVID-19	Influenza	p value¶		COVID-19	Influenza	
Total	4,139/21,615 (19.1)	678,845/2,380,696 (28.5)	<0.001		NA	NA	NA
Gastrointestinal disease	856/12,089 (7.1)	138,926/1,487,277 (9.3)	<0.001		11.39	14.57	0.78 (0.73–0.84)
Musculoskeletal disorder	772/9,712 (7.9)	104,663/1,512,773 (6.9)	<0.001		13.24	17.57	0.75 (0.70–0.81)
Periodontal disease	953/7,121 (13.4)	182,738/1,112,593 (16.4)	<0.001		15.61	24.02	0.65 (0.61–0.70)
Skin disease	797/14,638 (5.4)	153,928/1,358,895 (11.3)	<0.001		9.56	10.70	0.89 (0.82–0.98)
Hair loss	67/21,364 (0.3)	5,643/2,358,346 (0.2)	0.02		0.46	0.30	1.52 (1.18–1.97)
Asthma	99/20,372 (0.5)	61,699/1,990,519 (3.1)	<0.001		0.80	2.20	0.36 (0.28–0.47)
COPD	33/21,416 (0.2)	4,048/2,362,939 (0.2)	0.54		0.18	0.23	0.79 (0.54–1.15)
Pneumonia	419/20,189 (2.1)	82,460/1,895,100 (4.4)	<0.001		3.0	2.94	1.02 (0.90–1.16)
Cardiovascular disease	88/20,849 (0.4)	7,930/2,333,972 (0.3)	0.42		0.56	0.54	1.05 (0.83–1.32)
Heart failure	73/21,306 (0.3)	3,602/2,365,516 (0.2)	<0.001		0.34	0.18	1.88 (1.42–2.50)
Cerebrovascular disease	64/21,001 (0.3)	5,020/2,353,824 (0.2)	0.004		0.34	0.28	1.21 (0.91–1.60)
Autoimmune disease	119/20,759 (0.6)	13,813/2,307,629 (0.6)	0.64		0.87	0.84	1.03 (0.86–1.25)
Mood disorder	381/19,916 (1.9)	23,993/2,279,373 (1.1)	<0.001		2.80	1.61	1.73 (1.56–1.93)
Dementia	106/20,921 (0.5)	5,534/2,358,412 (0.2)	<0.001		0.40	0.21	1.96 (1.52–2.55)

*COPD, chronic obstruction pulmonary disease; COVID-19, coronavirus disease; NA, not applicable. †The numerator is the number of persons who experienced complications in the disease-free population and the denominator is the number of persons who did not have a history of the selected complication within a 3-y period before COVID-19 or influenza infection. The proportion is cumulative incidence. ‡Age-standardized incidence (cases per 1,000 person-months) for COVID-19 was divided by the standardized incidence rate for influenza and standardized to the 2020 population. §Rate ratios are standardized to the 2020 population. ¶χ^2^ tests were performed to assess the difference in proportion between COVID-19 and influenza patients.

In COVID-19 patients, when we analyzed the risk for COVID-19 complications by age, the 20–44-year age group had the highest odds ratio (OR) for complications (Table 3). NHIS beneficiaries had a lower risk for complications than MA recipients (OR 0.85, 95% CI 0.75–0.97). Patients in the provinces of Daegu and Gyeongsangbuk had an OR of 1.44 (95% CI 1.31–1.59) higher than those in other areas. Patients hospitalized with mild disease had a higher OR than ambulatory patients (OR 1.19, 95% CI 1.09–1.30). In influenza patients, when we analyzed the risk for influenza complications, OR for female patients was 1.04 (95% CI 1.04–1.05); the OR was lower in patients <19 years of age and 45–64 years of age than in those 20–44 years of age and higher in patients 65–74 years and >75 years of age. NHIS beneficiaries had a lower risk for influenza complications (OR 0.94, 0.95% CI 0.92–0.96) than did MA recipients. Patients whose CCI scores were 1–2 points (OR 1.08, 95% CI 1.08–1.09) or >3 points (OR 1.12, 95% CI 1.10–1.13) had higher risk than those with a CCI score of 0. Patients with hospitalized mild disease (OR 1.22, 95% CI 1.21–1.23) and patients with hospitalized severe disease (OR 1.36, 95% CI 1.29–1.43) had higher ORs than ambulatory patients (Table 3).

**Table 3:** Factors associated with incidence of complications in study of COVID-19 and influenza patients, South Korea*

Variable	Adjusted odds ratio (95% CI)†	
	COVID-19, n = 21,615	Influenza, n = 2,380,696
Female (referent: male)	1.03 (0.96–1.11)	1.04 (1.04–1.05)
Age, y (referent: 20–44 y)		
0–19	0.69 (0.59–0.80)	0.94 (0.94–0.95)
45–64	0.81 (0.74–0.88)	0.97 (0.96–0.98)
65–74	0.86 (0.76–0.96)	1.07 (1.05–1.08)
>75	0.86 (0.75–0.99)	1.26 (1.24–1.28)
National Health Insurance Service (referent: Medical Aid)	0.85 (0.75–0.97)	0.94 (0.92–0.96)
Region (referent: other areas)		
Seoul metropolitan area	0.90 (0.82–0.99)	1.03 (1.02–1.03)
Daegu and Gyeongsangbuk province	1.44 (1.31–1.59)	1.02 (1.01–1.03)
Charlson Comorbidity Index score (referent: 0)		
1–2	1.02 (0.94–1.11)	1.08 (1.08–1.09)
>3	1.01 (0.88–1.16)	1.12 (1.10–1.13)
Severity (referent ambulatory state)		
Hospitalized mild disease	1.19 (1.09–1.30)	1.22 (1.21–1.23)
Hospitalized severe disease	1.11 (0.91–1.34)	1.36 (1.29–1.43)

*COVID-19, coronavirus disease. †Multivariable logistic regression includes sex, age, type of insurance, region, and Charlson Comorbidity Index.

Female patients had higher risk than male patients for gastrointestinal disease (OR 1.37, 95% CI 1.19–1.58), musculoskeletal disorder (OR 1.31, 95% CI 1.12–1.52), periodontal disease (OR 1.23, 95% CI 1.06–1.41), and autoimmune disease (OR 2.11, 95% CI 1.40–3.19). Older patients (>65 years of age) had a higher risk for pneumonia, cardiovascular disease, heart failure, and cerebrovascular disease than young and middle-aged adults. Compared with MA recipients, NHIS beneficiaries had less dermatitis (OR 0.69, 95% CI 0.53–0.90), cardiovascular disease (OR 0.51, 95% CI 0.30–0.90), heart failure (OR 0.49, 95% CI 0.27–0.90), cerebrovascular disease (OR 0.40, 95% CI 0.22–0.73), autoimmune disease (OR 0.57, 95% CI 0.33–0.93), and dementia (OR 0.32, 95% CI 0.20–0.49). By region, the provinces of Daegu and Gyeongsangbuk had an increased risk of cerebrovascular disease (OR 2.11, 95% CI 1.02–4.37), mood disorder (OR 1.36, 95% CI 1.02–1.82), and dementia (OR 2.11, 95% CI 1.89–3.76). Hospitalized mild or severe patients had a higher risk for hair loss, pneumonia, and cardiovascular disease. The OR for pneumonia was 2.95 (95% CI 2.09–4.17) in patients 65–74 years of age and 4.25 (95% CI 2.96–6.12) in those >75 years. Cardiovascular disease risk was also higher for patients 65–74 years (OR 7.22, 95% CI 3.20–16.27) and for those >75 years of age (OR 5.15, 95% CI 2.11–12.60) (Appendix Table 2).

**Figure 2:**
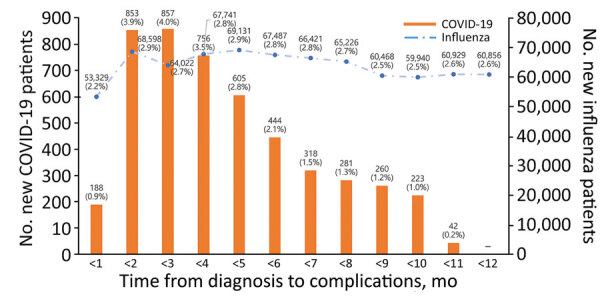
Time from diagnosis of COVID-19 or influenza to complications in study comparing complications in the 2 diseases, South Korea. COVID-19, coronavirus disease.

COVID-19 complications typically occurred 2–3 months after diagnosis (n = 1,711, 35.4%), and incidence within 1 month was 188 (3.9%). The incidence of influenza complications was high 2 months after diagnosis in 68,608 patients (9.0%), 1 month in 53,332 (7.0%), and 6 months in 373,853 (48.9%) (Figure 2; Appendix Table 3). 

## Discussion

In our study, COVID-19 typically had complication rates lower than or similar to those for influenza. However, the RRs for hair loss, heart failure, mood disorder, and dementia were marginally higher in the COVID-19 patient group. The incidence of COVID-19 complications was higher for patients 20–44 years of age, patients receiving MA, residents of the provinces of Daegu and Gyeongsangbuk, and patients with high CCI scores. In a subgroup analysis, the risk for hair loss was higher in younger adults, but the risk for mood disorders and dementia was higher in the elderly. Among those who had COVID-19, the elderly and the group with high CCI scores had more occurrences of pneumonia and cardiovascular disease; furthermore, the risk for cerebrovascular disease, mood disorder, and dementia was marginally higher in the provinces of Daegu and Gyeongsangbuk. We found that influenza patients were more likely to be younger than COVID-19 patients. Nevertheless, even after adjusting for age, complications were more common in influenza patients. Complications of COVID-19 that are more common than influenza complications are those that are more common in older adults. Although we adjusted for age, the effect of age may remain, and a stratified analysis by age is needed in future studies.

Various symptoms of COVID-19 persist even after recovery. In previous studies, general symptoms occur after COVID-19 infection, such as fatigue, sweating, and muscle pain; respiratory symptoms such as breathing difficulty and cough; psychosocial symptoms such as depression, anxiety, poor sleep quality, alopecia; and peripheral neuropathy, such as olfactory dysfunction (*14*–*18*). Because the natural course of COVID-19 is unknown, the types of long COVID are likely to increase (*19*,*20*). However, given that many persons are infected with COVID-19 globally or have recovered after being infected, it is necessary to assess the risk for long COVID-19.

In this study, 19.6% of COVID-19 patients had >1 complications. In South Korea, asymptomatic COVID-19 patients were discovered and managed, so almost all COVID-19 patients were included in the study. As a result, the complication rate from COVID-19 may be lower than that for other studies (*21*). Complications occurred more frequently in COVID-19 patients than in influenza patients and are consistent with previously known complications of COVID-19. Heart failure was more common in COVID-19 patients than in influenza patients. Both diseases have been reported to be associated with cardiovascular events because they are associated with inflammation. However, COVID-19 is associated with myocardial injury resulting from cytokine storm–related hyperinflammation and high expression of angiotensin-converting enzyme 2 receptors in myocardial tissue (*22*,*23*) that is thought to be followed by cardiomyopathy, arrhythmia, and heart failure (*24*,*25*). COVID-19 has proved to be an unprecedented long pandemic. Long-term social isolation causes loneliness, depression, and substance abuse (*26*,*27*) and alcohol consumption (*28*), and the rate of smoking increases during social isolation because of COVID-19 (*29*). In our study, we observed an increase in mood disorders; from our own and previously reported results, we inferred that the incidence rate may vary depending on the intensity of lockdown in each country. Hair loss is also a commonly reported symptom after COVID-19 infection (*16*,*30*). Although its pathogenesis is unclear, studies show that androgen increases COVID-19 susceptibility and enhances inflammation (*31*). Several reports of cerebrovascular disease have been reported after COVID-19 infection (*6*,*18*). The sequelae of cerebrovascular disease may have increased incidence of dementia; trends need to be monitored going forward.

This study has the advantage of confirming complications before and after diagnosis for all patients with COVID-19, influenza-like illness, or influenza who came to the hospital during the study period. South Korea conducted proactive testing for COVID-19, even for persons with no symptoms. Consequently, almost all COVID-19 patients, even those that were asymptomatic, were discovered and managed (*32*,*33*). Thus, the risk for selection bias was considered low. Moreover, because all residents were enrolled in the NHIS or MA, we were able to select patients who did not previously have the condition corresponding to the complication according to insurance data, increasing the accuracy of new complication detection. Influenza co-infection is possible among COVID-19 patients. However, in our study, only 259 (1.2%) patients had both COVID-19 and influenza, so we expected to see no significant effect.

Our study’s first limitation is that, because we identified the occurrence of complications from the claims data, the number of occurrences may have not been counted if the patient experienced mild or vague symptoms that did not prompt them to go to the hospital. Second, we recorded diagnoses that occurred after COVID-19 infection; however, we could not confirm a direct association with COVID-19. Third, it was difficult to determine the actual duration of symptoms; therefore, we could not clearly differentiate acute complications and long COVID. However, most symptoms occurred after 1 month; thus, they were considered long COVID. Fourth, only data through December 31, 2020, could be analyzed because of limitations in the claims data collection; therefore, we could not analyze complications caused by more recent SARS-CoV-2 variants, such as B.1.1.7 (Alpha) and B.1.351 (Beta). Further tracking of new complications is required. Monitoring with a prospective cohort that can be traced, including vague symptoms, is necessary.

In conclusion, although COVID is generally associated with fewer complications than influenza, caution is needed in groups with a high risk for hair loss, heart failure, mood disorder, dementia, and in patients with a high risk for complications, such as younger patients 20–44 years of age, and patients in COVID-19 hotspots. The risk for mood disorders and dementia was higher in the elderly. Although the complication rate of COVID-19 is not high, the fatality rate is of grave concern. If the fatality rate for COVID-19 is reduced through vaccination, the country can consider adopting less stringent COVID-19 management strategies, similar to those for influenza. Restrictions on economic activity or cross-border movement could be eased, and requirements for isolation of patients and close contacts could be modified. 

Appendix Additional information about the comparison of complications from coronavirus disease with complications from influenza, South Korea.
